# Enhancing Si_3_N_4_ Waveguide Nonlinearity with Heterogeneous Integration of Few-Layer WS_2_

**DOI:** 10.1021/acsphotonics.1c00767

**Published:** 2021-09-03

**Authors:** Yuchen Wang, Vincent Pelgrin, Samuel Gyger, Gius Md Uddin, Xueyin Bai, Christian Lafforgue, Laurent Vivien, Klaus D. Jöns, Eric Cassan, Zhipei Sun

**Affiliations:** † Department of Electronics and Nanoengineering, Aalto University, Espoo, 02150, Finland; ‡ Université Paris-Saclay, CNRS, Centre de Nanosciences et de Nanotechnologies, Palaiseau, 91120, France; § Department of Applied Physics, KTH Royal Institute of Technology, Stockholm, 114 28, Sweden; ∥ Institute for Photonic Quantum Systems (PhoQS), Center for Optoelectronics and Photonics Paderborn (CeOPP) and Department of Physics, Paderborn University, 33098 Paderborn, Germany; # QTF Centre of Excellence, Department of Applied Physics, Aalto University, Espoo, 02150, Finland

**Keywords:** low-dimensional materials, silicon photonics, integrated nonlinear optics, hybrid photonic waveguides, ultrafast optics

## Abstract

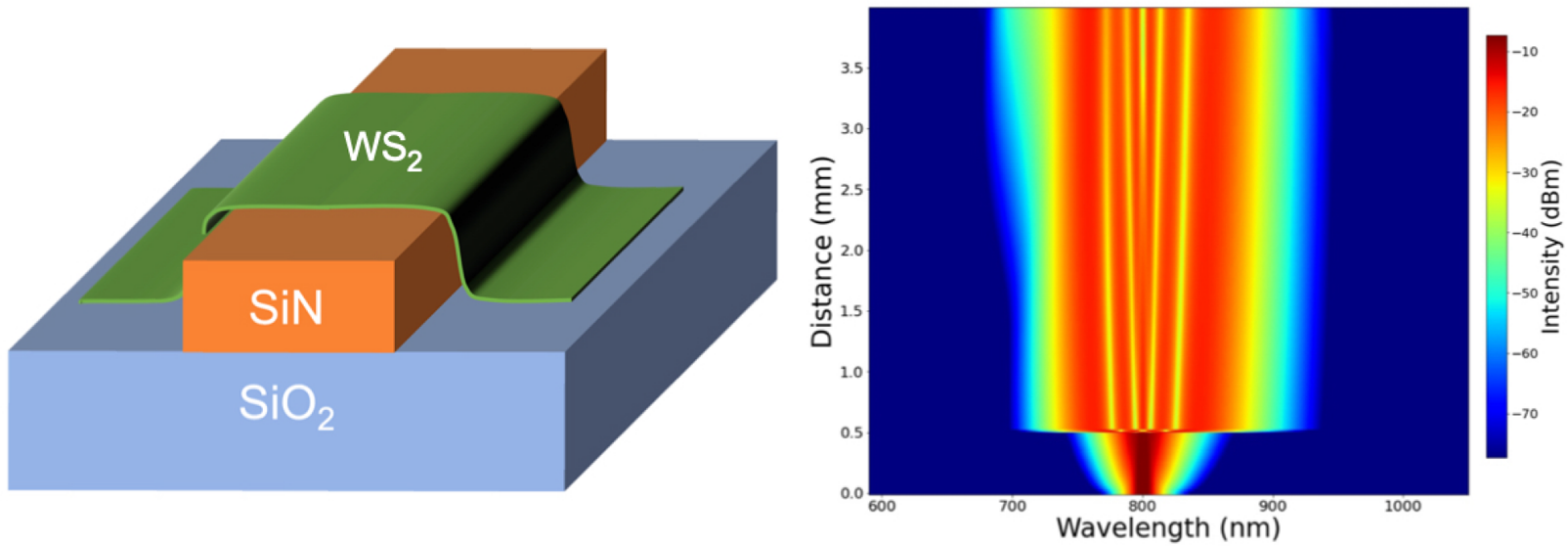

The heterogeneous integration of low-dimensional materials with photonic waveguides has spurred wide research interest. Here, we report on the experimental investigation and the numerical modeling of enhanced nonlinear pulse broadening in silicon nitride waveguides with the heterogeneous integration of few-layer WS_2_. After transferring a few-layer WS_2_ flake of ∼14.8 μm length, the pulse spectral broadening in a dispersion-engineered silicon nitride waveguide has been enhanced by ∼48.8% in bandwidth. Through numerical modeling, an effective nonlinear coefficient higher than 600 m^–1^ W^-1^ has been retrieved for the heterogeneous waveguide indicating an enhancement factor of larger than 300 with respect to the pristine waveguide at a wavelength of 800 nm. With further advances in two-dimensional material fabrication and integration techniques, on-chip heterostructures will offer another degree of freedom for waveguide engineering, enabling high-performance nonlinear optical devices, such as frequency combs and quantum light sources.

Following recent innovations in integrated optical frequency comb sources and their spectroscopy techniques, breakthroughs have been demonstrated in many fields of study, including precision spectroscopy,^1,2^ low-noise frequency synthesis,^3^ distance ranging,^4^ and quantum light sources.^5^ At the moment, two techniques are among the most frequently used for comb generation: the supercontinuum generation (SCG) process in nonlinear fibers or waveguides pumped with femtosecond mode-locked lasers and the four-wave mixing (FWM) process in microresonators pumped with continuous-wave lasers. The integration and the miniaturization of such versatile and powerful broadband coherent light sources with these two techniques have attracted tremendous endeavors, leading to great successes in the development of soliton microcombs,^6,7^ on-chip mode-locked lasers,^8^ and octave-spanning supercontinuum generation (SCG).^9^ It is worth mentioning that monolithic and low-cost chip-scale optical frequency combs are already on the verge of reality and envisioned to have a strong impact on both fundamental science and industrial applications.

To take advantage of mature infrastructures and technologies in the CMOS integrated circuit fabrication industry, silicon and silicon nitride (Si_3_N_4_, abbreviated as SiN) are considered among the most convenient materials for integrated photonic devices. Although silicon possesses excellent properties for on-chip comb generation based on both FWM and self-phase modulation (SPM) thanks to its strong third-order nonlinearity, it is limited in power-scaling by the two-photon absorption (TPA) and the free-carrier absorption (FCA) in the near-infrared (near-IR) region between ∼1.1 and 2 μm.^10^ The limitation due to FCA can be mitigated by the reverse-biasing of a waveguide integrated with a p-i-n junction.^11^ However, the limitation arising from TPA cannot be easily overcome. In comparison, SiN does not suffer from TPA in the same spectral region thanks to its larger bandgap, but it exhibits a weaker Kerr coefficient (*n*_2_ = ∼2.6 × 10^–19^ m^–2^ W^-1^ as compared to ∼4.5 × 10^–18^ m^–2^ W^-1^ for Si, at 1550 nm).^12^ Therefore, a trade-off needs to be taken into account while engineering these devices for optimized performance.

With rapidly increasing interest in two-dimensional (2D) layered materials, many of their peculiar properties have been observed and investigated. In particular, some 2D materials exhibit large nonlinearities, several orders of magnitude larger than conventional bulk nonlinear materials.^13−15^ Considering their minuscule dimensions down to the atomic level, they have great potential for integration with the SiN photonic platform for increasing its nonlinearities. Some of the most intensively studied 2D materials, such as graphene,^16−21^ graphene oxide,^22,23^ and MoS_2_,^24−26^ have been investigated for integration with silicon photonic waveguides to improve third-order nonlinear interactions, including SPM and FWM. Enhancements of FWM efficiency up to 7.3 dB in a SiN waveguide integrated with graphene oxide^22^ and 4 dB in a silicon waveguide integrated with MoS_2_^27^ have been successfully demonstrated. Recently, the heterogeneous integration of GaS^28^ thin-flakes with SiN microring resonators has been demonstrated, showing a 5-fold improvement of Kerr coefficient. In comparison to the aforementioned 2D materials, WS_2_ has an intriguing combination of optical properties, including good transparency and a strong nonlinearity in the near-infrared wavelength range, making it a potential choice for on-chip nonlinear optics, especially in the conventional telecom windows at ∼1.5 μm.^15,24,29^

In this Article, we report on the enhanced on-chip pulse broadening at 800 nm through SPM in a near-zero normal dispersion regime by the integration of few-layer WS_2_ on SiN waveguides. The resulting pulse full-width at half-maximum (fwhm) bandwidth has been improved by ∼48.8% using a few-layer WS_2_ flake of ∼14.8 μm in length. Based on the experimental results, a numerical model has been developed for the WS_2_–SiN heterogeneous waveguide structure, from which an enhancement in the waveguide effective Kerr coefficient of a factor of larger than 300 is retrieved at the wavelength of 800 nm. Thanks to the parallel orientation of the flake with the waveguide transverse electric (TE) mode, the in-plane Kerr coefficient of the few-layer WS_2_ is estimated from our mode overlap model to be ∼2.18 × 10^–15^ m^2^ W^-1^, which is comparable to previously reported values.^29^ These experimental results prove the intriguing potential of 2D material heterogeneous waveguides for low-loss and high-efficiency on-chip nonlinear optical circuits for frequency comb synthesis and entangled photon pair generation for quantum information processing.^5^

## Results and Discussion

**Figure 1:**
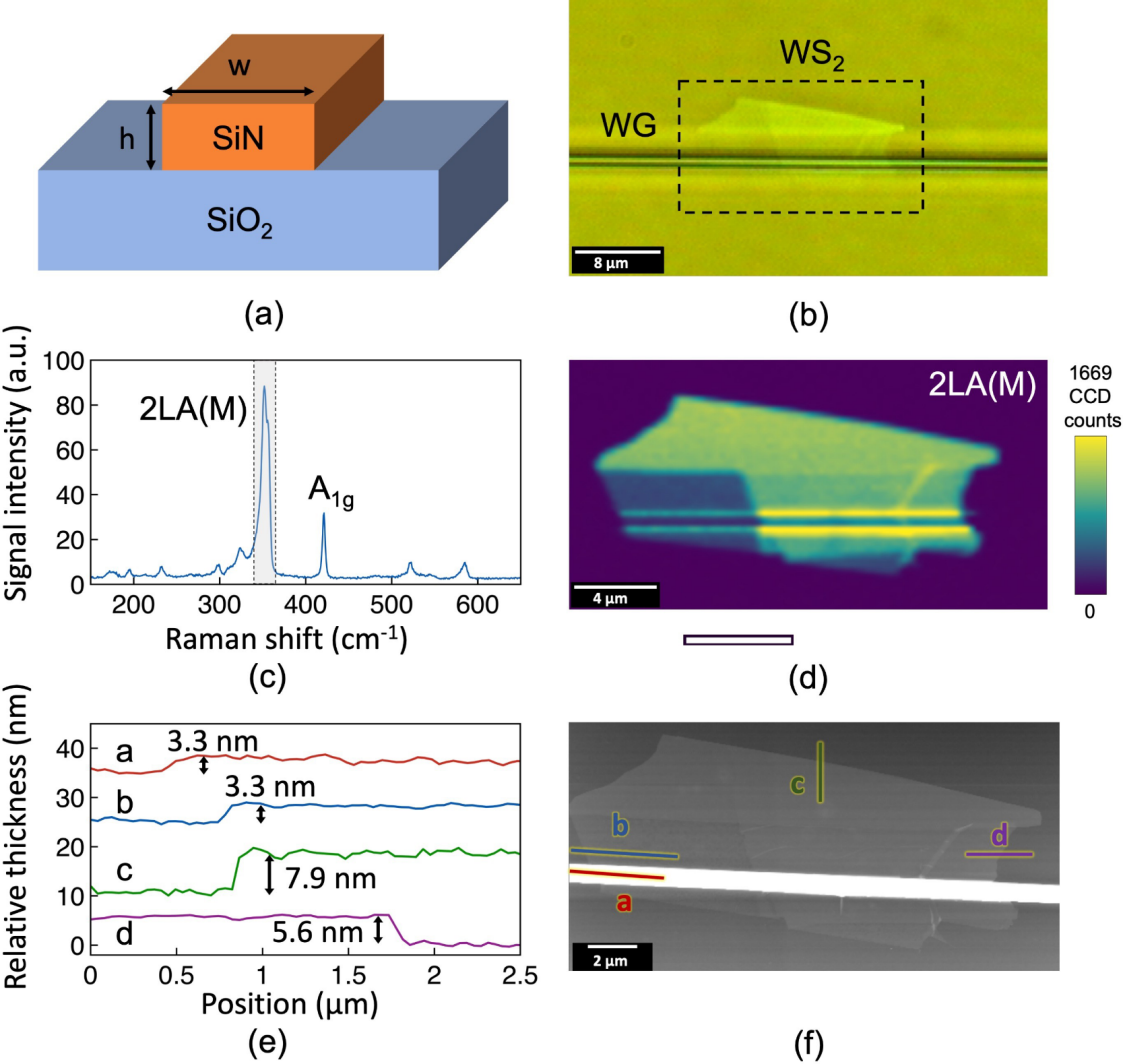
Heterogeneous waveguide structure: (a) the waveguide cross-section geometry, (b) the transferred WS_2_ flake on the waveguide (WG), (c) the Raman spectrum of the WS_2_ flake, (d) the Raman signal intensity map of the heterogeneous structure, integrated around the characteristic 2LA peak of WS_2_, (e) the relative thicknesses of the linear AFM scans along the marked paths, and (f) the AFM map of the heterogeneous waveguide with markings of paths for linear scans.

In this experiment, a few-layer WS_2_ flake is integrated on a ridged waveguide structure of 760 nm width (Figure 1a), as shown in the optical microscope image in Figure 1b. The heterogeneous structure is characterized with a scanning micro-Raman spectrometer. Using an excitation laser at 532 nm, the Raman mapping of the heterogeneous structure is performed with a spatial resolution of 500 nm. By summing the Raman spectra signals from ∼348 to 358 cm^–1^ and from ∼417 to 424 cm^–1^ (Figure 1c), the spectral intensities of the 2LA(M) peak and the A_1g_ peak of WS_2_ can be obtained, respectively. The integrated 2LA peak signal mapping is shown in Figure 1d. Consistently, it is clearly visible that the thicker the flake the more intense is the Raman signal. The thickness of the flake is also measured using an atomic force microscope (AFM), as shown in Figure 1e,f, where the waveguide has a height of 330 nm; therefore, it appears saturated in the grayscale image (Figure 1f). The flake covering the waveguide has a region of ∼3.3 nm thickness (∼6.5 μm overlap), a region of ∼7.9 nm thickness (∼6.3 μm overlap), and a region of ∼5.6 nm thickness (∼2 μm overlap). As a result, the average thickness is ∼5.57 nm, and the total overlap length is ∼14.8 μm.

**Figure 2:**
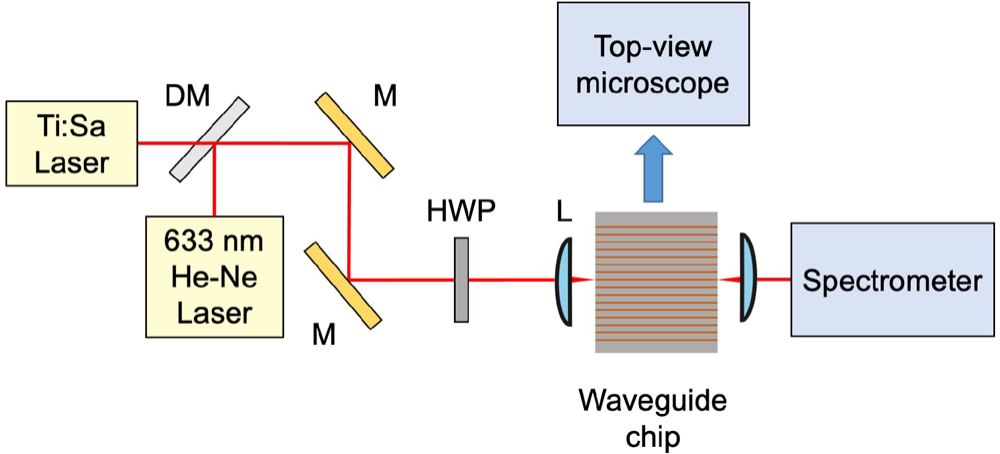
Schematics of the experimental setup for waveguide characterization: DM, dichroic mirror; M, gold mirrors; HWP, half-wave plate; L, aspheric lenses; Ti:Sa laser, Ti:sapphire laser.

The experimental setup of waveguide characterization is shown in Figure 2. A mode-locked Ti:sapphire laser operating at ∼84.5 MHz repetition rate with a pulse duration of ∼100 fs and a maximum average power of 300 mW is used as the pump source for nonlinear optical measurements. The pump power is controlled using a variable neutral density filter. The beam from the pump laser passes through a dichroic mirror (long-pass filter with a cutoff wavelength of 750 nm) and is then steered by a couple of gold mirrors before being focused by an aspheric lens of 1.45 mm focal length into the waveguides. A continuous-wave (CW) He–Ne laser emitting at 633 nm is aligned to the pump laser beam using the dichroic mirror to aid the waveguide coupling procedure and to perform passive waveguide loss measurements.

Using four spiral waveguides with different lengths on the same chip, the waveguide propagation loss is estimated to be around 1.12 dB/mm, while the coupling loss is retrieved to be around 5.86 dB (more details in the Supporting Information). With the addition of the WS_2_ flake, additional losses of ∼0.91 and 0.02 dB have been measured at 633 and 800 nm, respectively. The higher loss measured at 633 nm is attributed to the higher absorption coefficient of WS_2_ at that wavelength due to the linear absorption.^30^ Considering the overlap length of the flake, this leads to an additional propagation loss of ∼1.35 dB/mm at 800 nm. This result compares favorably to former reports of the additional propagation losses due to the integration of 2D materials in waveguide structures, for example, 2.05 dB/mm in a graphene oxide/silicon heterogeneous waveguide^23^ and 52 dB/mm in a graphene/silicon heterogeneous waveguide.^20^

**Figure 3:**
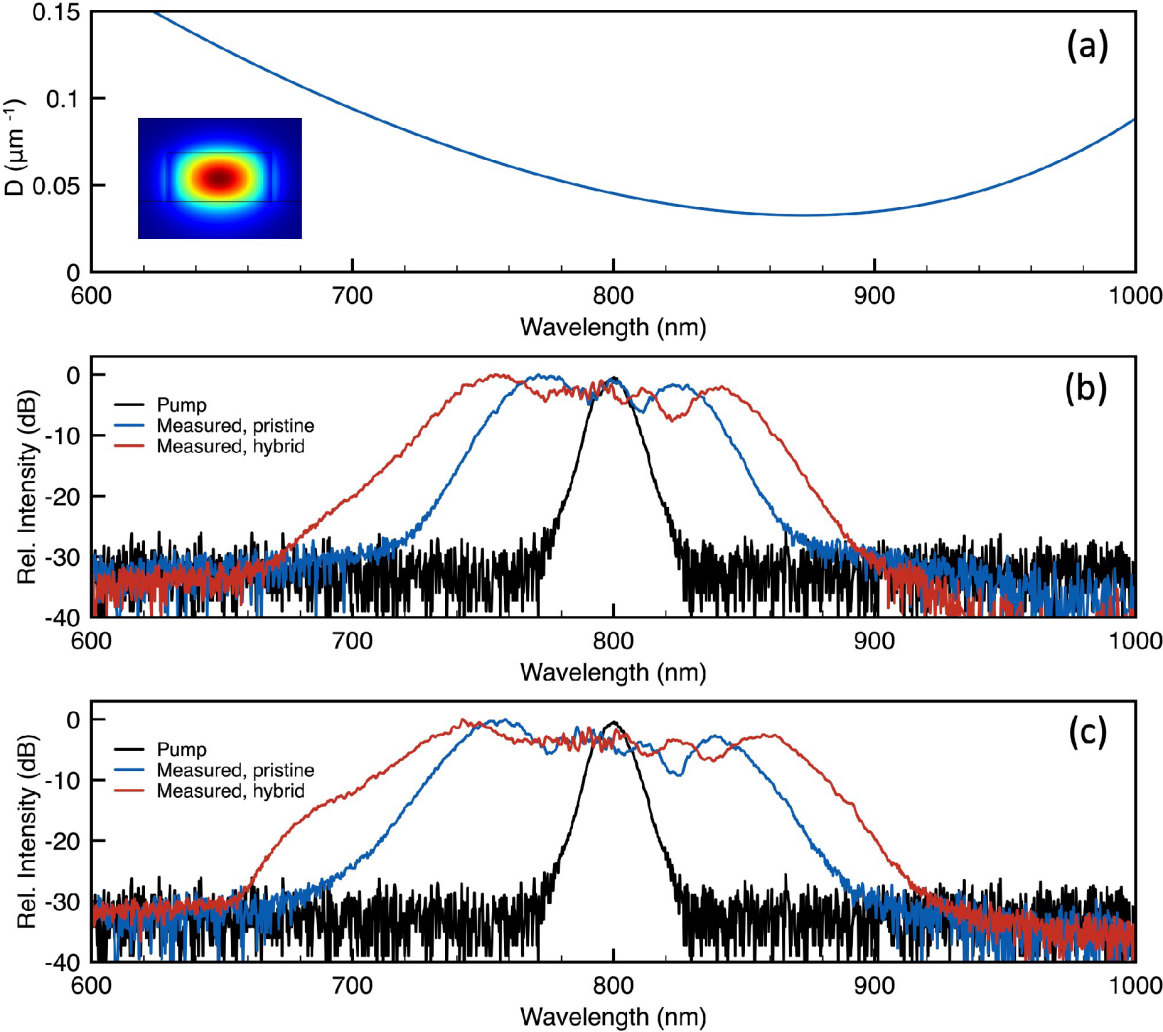
Dispersion properties and pulse broadening effects in the nonlinear waveguides. (a) Integrated dispersion of the TE mode of the pristine SiN waveguide with a width of 760 nm (inset: the calculated WG TE-mode profile at 800 nm); (b) measured pulse spectra of the pristine WG (blue) and the heterogeneous WG (red) at 1.35 kW pump peak power (input pump pulse spectrum shown in black); (c) measured pulse spectra of the pristine WG (blue) and heterogeneous WG (red) at 2.7 kW pump peak power (input pump pulse spectrum shown in black).

The nonlinear pulse propagation in the waveguides is first characterized without the integration of WS_2_. According to the design of the waveguide structure (details in Methods), the waveguide in this experiment has a minimum of integrated dispersion near to the pump wavelength of 800 nm (Figure 3a), which makes it an ideal choice for characterizing the pulse broadening through the SPM process. The integrated dispersion can be expressed as , where β_*k*_ is the *k*^th^ order dispersion.^31^ By controlling the orientation of a half-wave plate, the TE mode of the waveguide is excited (details in the Supporting Information). The pump pulse spectrum is characterized to have a fwhm of ∼13 nm. After the nonlinear propagation in the 4 mm long waveguide, the fwhm of the output pulses increased to ∼70.9 nm at an injected peak pump power of ∼1.35 kW (see Figure 3b; ∼123.6 nm at −20 dB level). Further increasing the injected peak pump power to ∼2.7 kW, the fwhm of the output pulses broadened to ∼97.9 nm (as shown in Figure 3c; ∼163.5 nm at −20 dB level).

The same waveguide is then used for the heterogeneous integration. After the successful deposition of the few-layer WS_2_ flake (Figure 1b), the heterogeneous WS_2_–SiN waveguide produces a much broader spectrum under the same excitation conditions. The comparison between the spectra before and after the WS_2_ integration at two different pump power levels is shown in Figure 3b,c. At ∼1.35 kW of pump peak power, the fwhm of the output pulses increases to ∼105.5 nm (∼177.3 nm at −20 dB level). At ∼2.7 kW of peak power, the spectrum is further broadened to a fwhm bandwidth of ∼133.5 nm (from ∼730.0 to 863.5 nm) and covering a bandwidth of ∼228.1 nm at −20 dB level (from ∼672.7 to 900.8 nm). As a result, with the addition of the few-layer WS_2_ flake, the fwhm of output pulses increased by ∼48.8% at ∼1.35 kW pump power and ∼36.4% at ∼2.7 kW pump power. These results translate into a maximum pulse broadening factor of ∼17.5 (WS_2_ covered waveguide pumped at ∼2.7 kW pulse peak power), the 13 nm wide input pulses broadens to ∼228 nm in the heterogeneous WS_2_–SiN waveguide of 4 mm total length. This is also a significant improvement compared to previously demonstrated on-chip pulse broadening/compression results.^32^

For a more straightforward comparison of the results, we introduce an enhancement factor *k* of the waveguide nonlinearity:
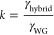
1where γ_hybrid_ is the effective nonlinear coefficient of the heterogeneous structure and γ_WG_ is the effective nonlinear coefficient of the pristine waveguide.

**Figure 4:**
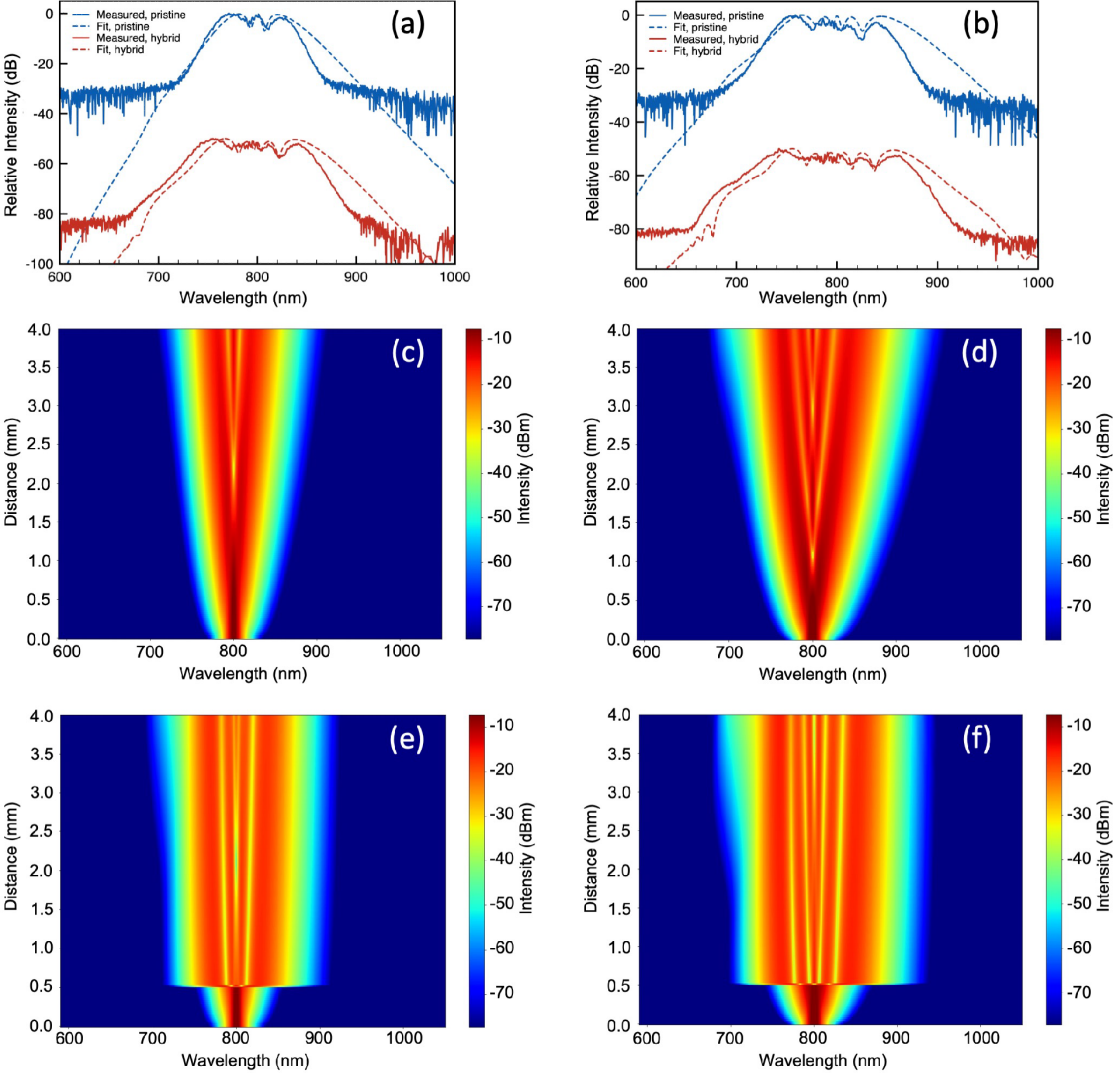
Simulated fitting curves for the pulse broadening in the waveguides: (a) measured pulse spectra (solid line) and simulated fitting spectra (dashed line) at 1.35 kW input peak pump power of the pristine WG (blue) and the heterogeneous WG (red); (b) measured pulse spectra (solid line) and simulated fitting spectra (dashed line) at 2.7 kW input peak pump power of the pristine WG (blue) and heterogeneous WG (red); and simulated pulse propagation false-color map of (c) the pristine waveguide at 1.35 kW input peak power, (d) the pristine waveguide at 2.7 kW input peak power, (e) the heterogeneous waveguide at 1.35 kW input peak power, and (f) the heterogeneous waveguide at 2.7 kW input peak power.

After constructing a numerical model for the heterogeneous waveguide, numerical simulations of the nonlinear pulse propagation in the heterogeneous waveguide are carried out for the best fitting of the experimental results (shown in Figure 4; more details in Methods). The model takes into account the measured dimension of the WS_2_ flake (with a length of ∼14.8 μm and a thickness of ∼5.57 nm) and its location along the waveguide (at roughly 0.5 mm from the input facet). By adjusting a set of two parameters, the effective nonlinear coefficient and the TPA coefficient of the heterogeneous waveguide, the output pulse spectra can be recreated with respect to the measured results. In Figure 4c–f, the false-color maps illustrate the evolution of the spectrum of the pulses traveling along the nonlinear waveguides. The enhancement effect introduced by the WS_2_ flake is rather evident considering the small flake length of 14.8 μm (which is located at *z* = 0.5 mm from the input). The large nonlinearity in the heterogeneous section largely anticipates the saturation of the nonlinear broadening effect which does not happen in the pristine SiN waveguide with a length of 4 mm.

At a pump power of 1.35 kW, an effective nonlinear coefficient γ_hybrid_ of ∼650 W^–1^ m^–1^ is retrieved for the heterogeneous waveguide. While at 2.7 kW pump power, the retrieved effective nonlinear coefficient is slightly lower at ∼600 W^-1^ m^–1^. The lower effective nonlinear coefficient retrieved at higher pump power is most likely due to the limitation of the broadening effect arising from the stronger linear absorption in WS_2_ at wavelengths close to 600 nm. For both power levels, the TPA coefficient of WS_2_ that provides the best fitting is ∼158 cm/GW, which agrees with the experimentally measured values of ∼525 ± 205 cm/GW reported in ref (33). As a result, the heterogeneous waveguide shows a significant improvement compared to the pristine SiN waveguide (γ_SiN_ = ∼1.75 W^–1^ m^–1^), with enhancement factors *k* of ∼371.4 and ∼342.9 for input pump powers of 1.35 and 2.7 kW, respectively. With the retrieved real and imaginary parts of the effective third-order nonlinear coefficient at 1.35 kW pump power, the nonlinear figure-of-merit of the heterogeneous waveguide is estimated to be ∼1.7 using the definition in ref (34).

The results from this experiment are summarized and compared with previously reported experimental results of 2D material heterogeneous WG structures in Table 1. Currently, the largest nonlinear coefficients in channel waveguides are demonstrated with graphene-Si heterogeneous structures, with effective nonlinear coefficients exceeding 1000 m^2^ W^-1^.^18,21^ However, graphene also introduces high propagation losses. On the contrary, as a dielectric, graphene oxide (GO) does not absorb linearly and shows a large Kerr coefficient a few orders of magnitude higher than Si.^35^ Although transition metal dichalcogenides, such as MoS_2_ and WS_2_, do not offer Kerr coefficients as high as GO, they provide better versatilities, such as the access to second-order nonlinearity with their monolayers and complex optoelectronic devices for their semiconducting nature. Thanks to the optimization of the waveguide dispersion profile and material properties, the pulse broadening effects obtained in the WS_2_–SiN heterogeneous waveguide in this work is much more significant than the MoS_2_–Si heterogeneous waveguide previously reported (spectral bandwidth of ∼2.2 nm at −20 dB level and a difference of ∼10% between the heterogeneous waveguide and the pristine waveguide).^36^ The effective nonlinear coefficients of the WS_2_–SiN structure in this work (γ_eff_ = ∼600 m^–1^ W^-1^) also reaches the level achieved with GO-Si/SiN structures (γ_eff_ = 167 m^–1^ W^–1^).^22^

**Table 1:** Summary of the Experimental Results of Nonlinear Pulse Broadening and the Numerically Retrieved Nonlinear Optical Properties of the Waveguide Structures^a^

structure	λ (nm)	*P*_p_ (kW)	fwhm (nm)	γ_eff_ (m^–1^ W^–1^)	*n*_2_ (m^2^ W^–1^)	*K*	α_2_ (cm/GW)	ref
pristine SiN	800	1.35	70.9	1.75	3.3 × 10^–20^	-	-	this work
		2.7	97.9			-	-	
WS_2_-SiN		1.35	105.5	650	9.5 × 10^–19^	371.4	158	
		2.7	133.5	600	8.8 × 10^–19^	342.8		
								
graphene-SiN	1550	-	-	2000–6400	-	∼10^3^	-	(21)
graphene-Si		-	-	-	2 × 10^–17^	3	-	(17)
		-	-	1542	-	9.8	-	(18)
GO-Si		-	-	-	9.6 × 10^–17^	16	-	(23)
GO-SiN		-	-	167	1.5 × 10^–17^	5.6	-	(22)
MoS_2_-Si		-	-	-	4.7 × 10^–15^	5.6	-	(26)
GaS-SiN		-	-	-	-	5	-	(28)

a λ, center wavelength of optical characterizations; *P*_p_, pump peak power; γ_eff_, effective nonlinear coefficient; *k*, enhancement factor of the effective nonlinear coefficient; α_2_, retrieved TPA coefficient; Si, silicon.

Using the waveguide mode overlap model and the waveguide effective susceptibility model described by eq 4 in the Methods, the Kerr coefficient of the WS_2_ flake can be retrieved. Under the condition of 1.35 kW pump power, the Kerr coefficient *n*_2_ of the few-layer WS_2_ flake is estimated to be ∼2.18 × 10^–15^ m^2^ W^–1^ at 800 nm. Instead, at 2.7 kW pump power, the estimated Kerr coefficient is lower, at ∼2.01 × 10^–15^ m^2^ W^–1^. These retrieved values are on the same level as the experimental results of ∼1.15 × 10^–15^ m^2^ W^–1^ measured at 532 nm using a vectorial two-wave mixing method.^29^

Assisted by the detailed numerical model for the heterogeneous waveguide and the potential for a fully fiber-based alignment-free experimental setup, the heterogeneous integration method could be extended to the characterization of the linear and nonlinear optical responses of low-dimensional materials and their on-chip nonlinear optical circuit applications. Without the requirement of any moving part and free-space alignment, it is more stable and efficient than the conventional z-scan method. While exciting the waveguide transverse magnetic mode, the out-of-plane components of optical properties can also be easily accessed, which is prohibited in the z-scan method at normal incidence.

In conclusion, we have demonstrated the enhancement of nonlinear pulse broadening in a SiN waveguide integrated with few-layer WS_2_. In a condition of normal dispersion with a near-zero GVD, the enhancement of waveguide Kerr nonlinearity has been characterized with a mode-overlap model. From the measured results, an enhancement in the effective third-order nonlinear coefficient of a factor of larger than 300 has been retrieved, as compared to the pristine SiN waveguide. The significant pulse broadening demonstrated in the heterogeneous waveguides could find immediate applications in on-chip SCG and pulse compression. With a mode overlap and waveguide effective susceptibility model, the Kerr coefficient of few-layer WS_2_ at 800 nm was retrieved to be ∼2.18 × 10^–15^ m^2^ W^–1^. With the rapid advance in the direct growth of transition metal dichalcogenides employing conventional chemical vapor deposition (CVD) techniques,^37^ the fabrication of heterogeneous waveguide structures can also be compatible with the existing CMOS manusfacturing technologies and infrastructures. With the precise control of the number of layers and the flake dimension in the direct CVD growth, the waveguide effective nonlinearity can therefore be engineered. The heterogeneous integration of 2D materials is shown to be a viable strategy for enhancing the nonlinearity of conventional SiN waveguide platform, therefore, increasing the efficiency of nonlinear frequency conversion processes such as SCG, FWM, and parametric down-conversion.

## Methods

### Fabrication of the Heterogeneous Waveguide

The waveguide chip used in this work is fabricated from a commercial low-pressure chemical-vapor-deposited (LPCVD) silicon nitride wafer with a nitride thickness of ∼330 nm and an underlying silicon oxide layer with a thickness of ∼3.3 μm (Rogue Valley Microdevices). The waveguides are patterned using electron beam lithography with negative resist then followed by fluorine-based reactive ion etching. The waveguides have a ridged cross-sectional structure, as illustrated in Figure 1a, with a height of ∼330 nm and a width ranging from 540 to 840 nm. The width of 760 nm is then chosen after experimental confirmation, for an optimized dispersion profile. A s-shape displacement is made in the middle of the waveguide by two 90° turns, with a radius of curvature of 50 μm, to reduce stray light collection at the output end.

For the heterogeneous integration of WS_2_ few-layers with high optical quality, the mechanical exfoliation method is used for sample preparation. Mediated by polydimethylsiloxane stamps, exfoliated thin flakes are deterministically transferred onto the upper waveguide surface using a commercial nanomaterial transfer system (HQ Graphene). The entire exfoliation and transfer processes are performed in a cleanroom environment to avoid any contamination from undesired particles. By controlling the exfoliation process, a few-layer WS_2_ flake is isolated and then transferred on top of the waveguide without contaminating nearby waveguides.

### Measurement Apparatus

For the pulse broadening measurements, a Ti:sapphire femtosecond laser (Spectra-Physics MaiTai) emitting pulses at 800 nm central wavelength operating at a repetition rate of ∼84.5 MHz with a pulse duration of ∼100 fs (∼13 nm fwhm bandwidth) at a maximum average power of ∼300 mW is used as the pump source. The laser powers are measured with a photodiode-based power meter (Ophir) while the spectra of the laser pulses before and after nonlinear propagation are measured with a high-sensitivity spectrometer covering the visible to near-IR range (Ocean Optics HR 4000).

### Numerical Modeling

For a better understanding of the physical processes behind the nonlinear pulse propagation and the enhancement of the waveguide effective nonlinearity by introducing the WS_2_ flake, we have conducted detailed mode overlap analyses and nonlinear pulse propagation simulations.

The heterogeneous waveguide structure was analyzed using a commercial finite difference eigenmodes mode solver (Lumerical). The effect of the introduced WS_2_ flake can be modeled using its surface conductivity σ_s_ and relative permittivity ϵ_r_.^38^ The relative permittivity can be expressed as the sum of *N* Lorentzian functions:
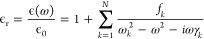
2where γ_*k*_ is the oscillator strength, ω_*k*_ is the resonance frequency, and *f*_*k*_ the spectral width, of the *k*^th^ oscillator. These coefficients used in this expression can be extracted from experimental measurements of WS_2_.^39^ Then the surface conductivity can be retrieved using the following expression of the dielectric function:
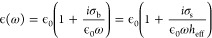
3where σ_b_ is the bulk conductivity of the material and *h*_eff_ is the effective thickness of the WS_2_ flake under consideration (∼5.57 nm).

After constituting the waveguide cross-section model using the calculated surface conductivity of WS_2_ and the Sellmeier equations for LPCVD Si_3_N_4_ and SiO_2_,^25,40^ the waveguide effective indices are calculated for the TE mode over a wide wavelength range from 600 to 1000 nm. It has been shown that compared to a waveguide with the same geometry without the 2D material, the material introduced little change to the waveguide dispersion.^41^ Therefore, here we assume that the heterogeneous waveguide has the same dispersion operator of the SiN waveguide before the integration of WS_2_ flake. The calculated total dispersion operator of a waveguide with a width of 760 nm is shown in Figure 3a. On the other hand, considering the thickness of the WS_2_ of ∼5.57 nm, the mode field distribution of the heterogeneous waveguide with the WS_2_ flake can be calculated. Here we consider the average thickness because the third-order nonlinearity of WS_2_ is shown to depend relatively linearly with the thickness (number of layers).^29^ As a result, an overlap factor of the guided mode with the WS_2_ flake is calculated to be η = 0.45% using the model reported in ref (41). In the end, an effective nonlinear susceptibility of the heterogeneous waveguide can be written as the weighted integral of the corresponding tensor susceptibility over the waveguide mode:^42^
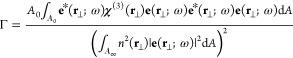
4where *A*_0_ is the area of the waveguide cross-section, *A*_∞_ denotes the integration to be performed across the entire transverse plane, ω is the frequency of the pump light, **e**(**r**_⊥_; ω) is the electric field profile of the waveguide mode (for our waveguide, which is uniform along its length, the mode depends only on the transverse coordinate **r**_⊥_), χ^(3)^(**r**_⊥_) is the third-order nonlinear susceptibility tensor over the transverse coordinate, and *n*(**r**_⊥_) is the refractive index over the transverse coordinate.

Using the calculated waveguide dispersion profile, the nonlinear pulse propagation in the waveguides has been modeled with the generalized nonlinear Schrödinger equation in the frequency domain.^31,43,44^ To better assess the influence of the WS_2_ flake to the SiN waveguide, the finite length of the flake has been taken into account. We divide the device into three sections: one heterogeneous section with a WS_2_ flake between two pristine SiN waveguides. For the simulation of spectral broadening, we use a transform-limited pump pulse with a duration of ∼100 fs and peak powers close to the estimated power coupled to the waveguide TE mode. Since the TPA coefficient is quite large for WS_2_ in the wavelength range of this experiment, we also take into account the TPA effect in the simulation.^33^ The SiN has an energy bandgap more than twice the pump photon energy, which is confirmed by the negligible TPA coefficient of 2.9 × 10^–8^ cm/GW measured in our experiment (see Supporting Information). Therefore, in the numerical model, we consider only the TPA coefficient of the WS_2_ flake. From the simulation results, reasonable fits to the experimental data can be obtained for both the simple waveguide and the heterogeneous waveguide, especially the peak wavelengths of the spectral features created by SPM in the normal dispersion regime (Figure 4a,b). At the longer wavelength side, the simulation departs slightly from the measured spectrum, most likely due to the limited accuracy in the dispersion profile. From the best fitting results, we can retrieve the effective nonlinear coefficient and the TPA coefficient of the heterogeneous waveguide.

## Supporting Information Available

Additional details of waveguide cross section scanning electron microscope images, WS_2_ flake characterization, waveguide loss characterization, and waveguide two-photon absorption characterization (PDF)
