# Supplementary material for: Enhancing Si3N4 Waveguide Nonlinearity with Heterogeneous Integration of Few-Layer WS2
Source: ACS Photonics. 2021 Sep 3;8(9):2713–21. doi: 10.1021/acsphotonics.1c00767 (PMC8447258; doi:10.1021/acsphotonics.1c00767)
Supplement: Supplementary file 1 — ph1c00767_si_002.pdf [file ph1c00767_si_002.pdf]

Supporting Information: Enhancing Si_3N_4 waveguide nonlinearity with heterogeneous integration of few-layer WS_2

Yuchen Wang,[*,†,⊥] Vincent Pelgrin,[†,‡,⊥] Samuel Gyger,[¶] Gius Md Uddin,[†] Xueyin Bai,[†] Christian Lafforgue,[‡] Laurent Vivien,[‡] Klaus D. Jöns,[§] Eric Cassan,[‡] and Zhipei Sun[*,†,‖]

†*Department of Electronics and Nanoengineering, Aalto University, Espoo, 02150, Finland*

‡*Université Paris-Saclay, CNRS, Centre de Nanosciences et de Nanotechnologies, Palaiseau, 91120, France*

¶*Department of Applied Physics, KTH Royal Institute of Technology, Stockholm, 114 28, Sweden*

§*Department of Physics, Paderborn University, Paderborn, 33098, Germany*

‖*QTF Centre of Excellence, Department of Applied Physics, Aalto University, Espoo, 02150, Finland*

⊥*Contributed equally to this work*

E-mail: yuchen.xindi.wang@gmail.com; zhipei.sun@aalto.fi

Characterization of the heterogenous waveguide structure

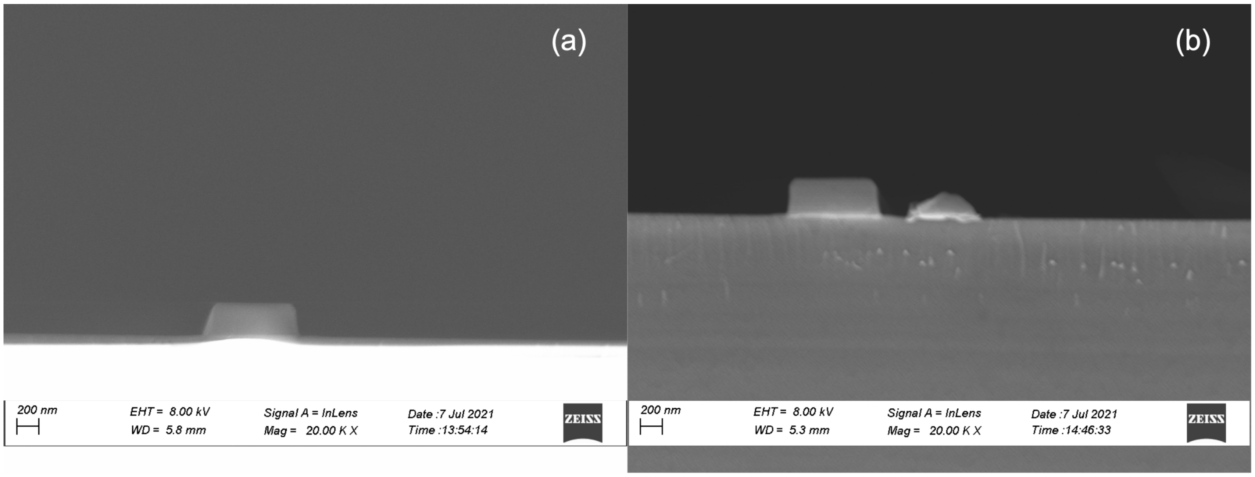

Figure S1: Scanning electron microscope (SEM) images of: (a) the silicon nitride waveguide with 760 nm width before gold coating and (b) the waveguide with 760 nm width after gold coating.

The waveguide cross section is characterized with a scanning electron microscope (Zeiss Sigma VP) to assess the quality of waveguide cross-section. The SEM images of the waveguide with 760-nm width and 330-nm height are shown in Fig.S1. As silicon nitride is an insulating dielectric material, a 4-nm thick gold layer was coated to the waveguide cross-section using physical vapor deposition method to improve the quality of the SEM image. In this additional process, some particles have been introduced onto the waveguide surface as seen in Fig.S1(b) which was not present before the sputtering (see Fig.S1(a)). With the effort in the optimization of fabrication processes of the SiN ridge waveguides, the waveguide sidewalls and the top surface are visibly smooth, which contribute to the relatively low propagation losses measured in these waveguides.

The dimension of the flake is fundamental to the analysis of its influence on the waveguide nonlinearity. We performed the length measurement of the flake using an optical microscope with a 100x objective lens (Olympus BX60) before the transfer process. The length of the flake overlapping with the waveguide is measured to be 14.8 µm. The thickness of the flake has been measured using an atomic force microscope (Bruker). A mapping of the thickness

of the flake covering the hybrid waveguide is shown in Fig. 1(f) in the main text. The flake covering the waveguide has a region of ~3.3 nm thickness (~6.5 µm overlap), a region of ~7.8 nm thickness (~6.3 µm overlap) and a region of ~5.6 nm thickness (~2 µm overlap). As a result, the average thickness is ~5.57 nm and the total overlap length is ~14.8 µm.

Characterization of the losses in the SiN waveguide

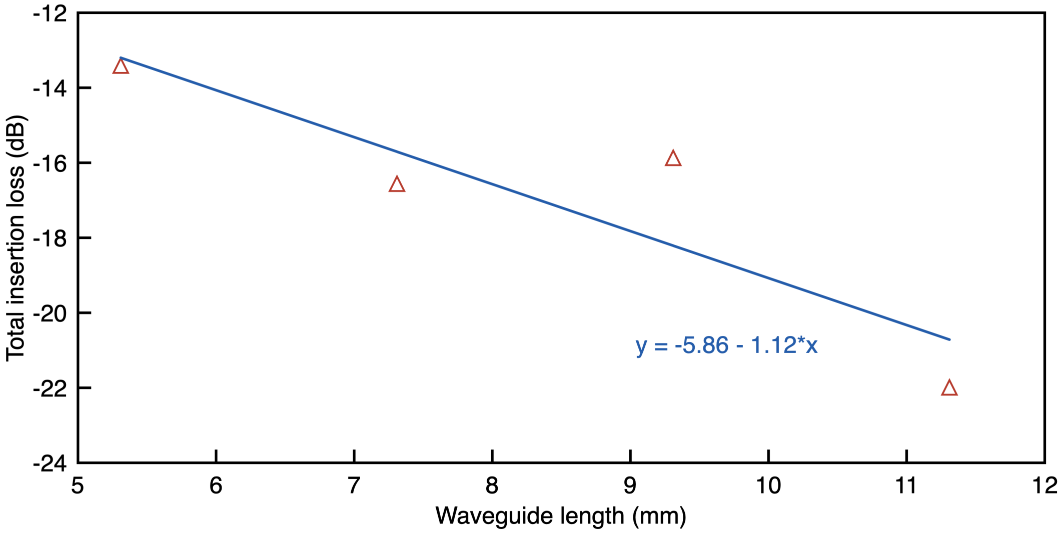

Figure S2: Waveguide propagation loss characterization. The red triangles represent the measured total insertion losses of waveguides with 800 nm width and different lengths. The blue line represents a linear regression of the logarithmic loss.

The waveguide insertion loss is characterized using mainly the continuous-wave He-Ne laser. Because of the irregularities of the chip end surface and the varying waveguide widths, the coupling efficiency varies between waveguides. For a reliable estimation of the waveguide propagation loss and the coupling efficiency of the setup, we used a set of 800 nm wide waveguides with different lengths on the same chip as the hybrid waveguide.

The losses can be attributed mainly to two components: the coupling loss η_c which is a constant and the propagation loss γ_p which scales with the propagation length l. The coupling loss includes the mode matching efficiency and other fixed losses such as the loss due to the Fresnel reflection. The propagation loss includes losses due to absorption and scattering

along the waveguide. As a result, the total insertion loss γ (in dB) of the waveguide can be expressed as the sum of all logarithmic loss terms:

$$\gamma = \eta_c + \gamma_p, \tag{1}$$

From the experimental measurements, we retrieve a coupling loss of -5.86 dB and a propagation loss of -1.12 dB/mm (as shown in Fig. S2). Before and after the transfer of the WS_2 flake to a waveguide with a width of 760 nm, the same measurement has been performed with an excitation wavelength of 632 nm showing an additional loss of -0.9 dB due to the flake. This additional loss reduces to a negligible level (-0.02 dB) when an excitation wavelength of 800 nm was used. The higher loss of the hybrid waveguide at 632 nm could be due to the higher absorption coefficient of WS_2 at the shorter wavelength.

Characterization of the two-photon absorption in the SiN waveguide

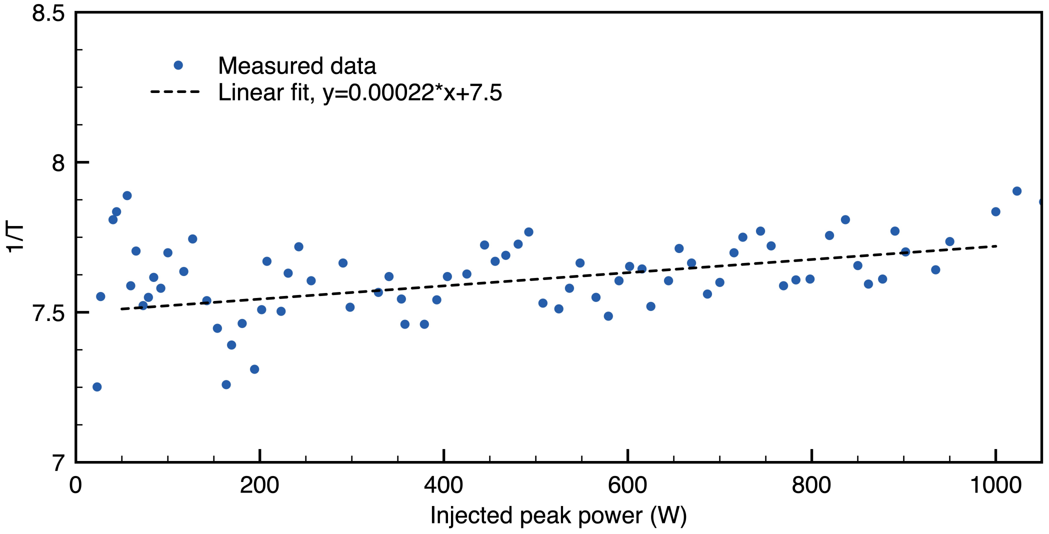

Figure S3: Measured waveguide inverse transmission versus the injected peak pump power. T, transmission of the waveguide.

For a better understanding of the losses in the silicon nitride waveguide, we have per-

formed power-dependent waveguide transmission measurements. For the waveguide in this experiment with a width of 700 nm, the overall input-to-output transmission has been measured from 1 mW to 30 mW average pump power. The inverse transmission (1/T) is plotted against the injected peak power in Fig. S3. The values of T are the measured total output power divided by the injected pump power.

From the experimental results we can retrieve the TPA coefficient β_2 according to method reported in Ref.,[S1] using the following expression:

$$\frac{P_{in}}{P_{out}} = \exp(\alpha L)\frac{L_{eff}}{A_{eff}}\beta_2 P_{in} + \exp(\alpha L), \tag{2}$$

where P_{in} and P_{out} are input and output powers, α is the absorption coefficient, $L_{eff} = (1 - \exp(-\alpha L))/\alpha$ is an effective propagation length, A_{eff} is the effective area of waveguide mode. From our measurements, the TPA coefficient of SiN at 800 nm is estimated to be 2.9×10^{-8} cm/GW. This is a negligible value as expected, as the photon energy at 800 nm (1.55 eV) is well below half of the energy bandgap of amorphous SiN (half of 4.6 to 5.2 eV).

Acknowledgement

The authors acknowledge funding from Academy of Finland (314810, 333982, 336144 and 336818), Academy of Finland Flagship Programme (320167,PREIN), the European Union's Horizon 2020 research and innovation program (820423,S2QUIP), the EU H2020-MSCA-RISE-872049 (IPN-Bio), and European Research Council (647342, 834742) and Agence Nationale de la Recherche (ANR OpticAll).

References

(S1) Tsang, H.; Penty, R.; White, I.; Grant, R.; Sibbett, W.; Soole, J.; LeBlanc, H.; Andreadakis, N.; Bhat, R.; Koza, M. Two-photon absorption and self-phase modulation

in InGaAsP/InP multi-quantum-well waveguides. *J. Appl. Phys.* **1991**, *70*, 3992–3994.
